# Commonly applied positive end-expiratory pressures do not prevent functional residual capacity decline in the setting of intra-abdominal hypertension: a pig model

**DOI:** 10.1186/cc9095

**Published:** 2010-07-02

**Authors:** Adrian Regli, Lisen E Hockings, Gabrielle C Musk, Brigit Roberts, Bill Noffsinger, Bhajan Singh, Peter V van Heerden

**Affiliations:** 1 Intensive Care Unit, Sir Charles Gairdner Hospital, Hospital Avenue, Nedlands (Perth) WA 6009, Australia; 2 Veterinary Anaesthesia, Murdoch University Veterinary Hospital, 90 South Street, Murdoch (Perth) WA 6150, Australia; 3 Department of Pulmonary Physiology, Sir Charles Gairdner Hospital, Hospital Avenue, Nedlands (Perth) WA 6009, Australia

## Abstract

**Introduction:**

Intra-abdominal hypertension is common in critically ill patients and is associated with increased morbidity and mortality. The optimal ventilation strategy remains unclear in these patients. We examined the effect of positive end-expiratory pressures (PEEP) on functional residual capacity (FRC) and oxygen delivery in a pig model of intra-abdominal hypertension.

**Methods:**

Thirteen adult pigs received standardised anaesthesia and ventilation. We randomised three levels of intra-abdominal pressure (3 mmHg (baseline), 18 mmHg, and 26 mmHg) and four commonly applied levels of PEEP (5, 8, 12 and 15 cmH_2_O). Intra-abdominal pressures were generated by inflating an intra-abdominal balloon. We measured intra-abdominal (bladder) pressure, functional residual capacity, cardiac output, haemoglobin and oxygen saturation, and calculated oxygen delivery.

**Results:**

Raised intra-abdominal pressure decreased FRC but did not change cardiac output. PEEP increased FRC at baseline intra-abdominal pressure. The decline in FRC with raised intra-abdominal pressure was partly reversed by PEEP at 18 mmHg intra-abdominal pressure and not at all at 26 mmHg intra-abdominal pressure. PEEP significantly decreased cardiac output and oxygen delivery at baseline and at 26 mmHg intra-abdominal pressure but not at 18 mmHg intra-abdominal pressure.

**Conclusions:**

In a pig model of intra-abdominal hypertension, PEEP up to 15 cmH_2_O did not prevent the FRC decline caused by intra-abdominal hypertension and was associated with reduced oxygen delivery as a consequence of reduced cardiac output. This implies that PEEP levels inferior to the corresponding intra-abdominal pressures cannot be recommended to prevent FRC decline in the setting of intra-abdominal hypertension.

## Introduction

Intra-abdominal hypertension (IAH) is defined by the World Society of Abdominal Compartment Syndrome as a sustained increase in intra-abdominal pressure (IAP) above or equal to 12 mmHg and abdominal compartment syndrome is defined as an IAP of more than 20 mmHg plus a new organ dysfunction [1]. IAH and abdominal compartment syndrome are common in critically ill patients and are associated with a high rate of morbidity and mortality [1-6]. IAH is associated with an increased systemic vascular resistance, a decreased venous return and a reduced cardiac output subsequently leading to reduced renal, hepatic and gastro-intestinal perfusion and thereby promoting multi organ failure [7-12].

Patients with IAH are susceptible to a significant impairment in lung function mainly caused by atelectasis resulting from a cephaled shift of the diaphragm, with subsequent decrease in lung volume leading to a decrease in arterial oxygenation [12-14]. Atelectasis is generally treated by recruitment manoeuvres followed by increasing positive end expiratory pressure (PEEP) in patients receiving mechanical ventilation [14-17]. However, in the setting of IAH, the role of PEEP remains unclear. On one hand increased levels of PEEP have been proposed to improve lung function [13,18]. On the other hand low levels of PEEP have been suggested to avoid haemodynamic compromise [7].

The correct diagnosis and treatment of the underlying condition and, where medical treatment fails and as a last resort, the performance of a decompressive laparotomy is recommended in patients with severe IAH (> 25 mmHg) [2]. However, in patients with less severe IAH or prior abdominal surgery in patients with severe IAH, the World Society of Abdominal Compartment Syndrome recommends that cardiac output (CO) and oxygen delivery (DO_2_) should be optimized, as this has been associated with a reduced morbidity and mortality in these patients [2,8].

The aim of this project was to study the effect of commonly applied PEEP levels on FRC, arterial oxygen saturation, CO and DO_2 _in a healthy pig model of IAH. We hypothesized that PEEP would increase FRC and decrease CO and that there would be a PEEP level at which DO_2 _would be optimal. We also hypothesized that high levels of PEEP would increase IAP.

## Materials and methods

The study conformed to the regulations of the Australian code of practice for the care and use of animals for scientific purposes and was approved by the Animal Ethics Committee of the University of Western Australia.

### Preparation of animals and anaesthesia

We studied 13 pigs (Large White breed), which were fasted overnight, but with free access to water. Each of the animals was weighed and then sedated with an intramuscular injection of Zoletil^® ^(1:1 combination of tiletamine and zolazepam, Virbac, Milperra, NSW, Australia) (4 mg/kg) and xylazine (2 mg/kg). Venous access was then established and secured in an auricular vein. To facilitate endotracheal intubation, an intravenous (IV) bolus of propofol (1 mg/kg) was administered. The trachea was intubated via the oral route with a cuffed endotracheal tube (size 8.0 mm, Hi-Lo, Mallinckrodt, Athlone, Ireland). Anaesthesia was maintained with a combination of propofol (9 to 36 mg/kg/h IV), morphine (0.1 to 0.2 mg/kg/h IV) and ketamine (0.3 to 0.6 mg/kg/h IV) according to clinical requirements. Neuromuscular blocking agents were not administered. A core temperature of 36°C to 38°C was maintained by the application of heating mats.

Succinylated gelatin (Gelofusine, Braun, Oss, The Netherlands) was given pre-emptively for haemodynamic stabilization (500 mL over the first 30 minutes followed by 1 mL/kg/h). At the end of the protocol the pigs were euthanized with pentobarbitone (100 mg/kg body weight), injected IV.

### Ventilation

A critical care ventilator (Servo 900, Siemens, Berlin, Germany) was used with the following ventilator settings: FiO_2 _0.4, volume control mode, I:E ratio of 1:2, tidal volume of 6 ml/kg with the respiratory rate adjusted to maintain an end tidal CO_2 _of 35 to 45 mmHg. The initial PEEP setting was 5 cmH_2_O (3.7 mmHg) and altered according to the experimental protocol. Peak airway pressure (pPaw), mean airway pressure (mPaw), and dynamic compliance (Cdyn) were measured by the ventilator.

### Surgical procedure

Throughout the study the animals remained supine. Following a chlorhexidine based antiseptic skin preparation the pigs were instrumented as follows:

#### Haemodynamic monitoring

A 16-gauge single lumen catheter (ES-04301, Arrow International, Reading, PA, USA) was inserted into the femoral artery to measure the mean arterial blood pressure (MAP). An 8.5F percutaneous introducer (SI-09806, Arrow International) was inserted into the internal jugular vein to allow the placement of a pulmonary artery catheter (AH-05050, Arrow International) under continuous pressure wave monitoring into the pulmonary artery in order to measure CO.

#### Intra-abdominal pressure measurement and generation

For the measurement of IAP, a caudal midline laparotomy was performed to place a 12F Foley catheter (226512, Bard, Covington, GA, USA) in the urinary bladder.

For the generation of different levels of IAP, we performed another cephaled midline laparotomy in order to place a latex balloon (200 g weather balloon, Scientific Sales, Lawrenceville, NJ, USA) in the peritoneal cavity. The abdomen was tightly closed with sutures. Inflation of the intra-abdominal balloon with air allowed the generation of different levels of IAP [19].

We measured IAP using urinary bladder pressure as defined by the World Society of Abdominal Compartment Syndrome with the only difference that we measured mean IAP instead of end-expiratory IAP [1]. We used a standardised injection volume of normal saline (25 ml syringe with auto-valve, AbViser, Wolfe Tory Medical, Salt Lake City, UT, USA). We measured urinary bladder pressure before and after alterations of PEEP.

### Experimental protocol

After a set of baseline measurements, the abdominal balloon was either not inflated (baseline IAP) or inflated with air to produce grade II (18 +/- 2 mmHg) or grade IV (26 +/- 2 mmHg) IAH in predefined random order [1]. PEEP was then applied in a predefined random manner at 5, 8, 12 or 15 cmH_2_O (3.7, 5.9, 8.8, and 11.0 mmHg, respectively) at each level of IAP; these are commonly used levels of PEEP in critically ill patients. For randomisation, we used a spit plot design ensuring all 12 combinations of IAH and PEEP levels were applied to all animals [20].

For each IAP and PEEP setting, we performed a standardised lung recruitment manoeuvre as follows [21]. PEEP was increased every respiratory cycle by increments of 2 cmH_2_O (1.5 mmHg) in order to achieve either a PEEP value of 15 cmH_2_O (11.0 mmHg) or a maximum peak airway pressure of 40 cmH_2_O (29.4 mmHg) and then continued for 10 consecutive breaths. Thereafter the PEEP was decreased by 2 cmH_2_O (1.5 mmHg) decrements per respiratory cycle until the target PEEP setting was achieved according to the experimental protocol. All respiratory and haemodynamic measurements were then performed after a five-minute period allowing for abdominal, respiratory and haemodynamic stabilization.

### Measurements and calculations

#### Haemodynamic parameters

All pressures including IAP were measured with a transducer (Hospira, Lake Forest, IL, USA) and monitored with a critical care monitor (Sirecust 126; Siemens Medical Electronics, Danvers, MA, USA). MAP, central venous pressure (CVP) and heart rate (from electro-cardiogram) were measured. All pressures were zeroed at the mid axillary line, including urinary bladder pressure [1]. CO was measured by thermodilution using a standardised 10 ml bolus of ice cold normal saline (Sirecust 126; Siemens Medical Electronics). For each IAP and PEEP setting, three CO measurements were performed and averaged.

#### Functional residual capacity

FRC was measured using the multiple breath nitrogen wash-out method [22]. After switching FiO_2 _from 0.4 to 1.0 an air tight bag collected the expiratory gas from the ventilator until < 0.5% nitrogen was detectable. The total expired gas volume was measured using a digital pneumotachograph (HP 47303A, Hewlett-Packard, Paranus, NJ, USA) and nitrogen concentration was measured with a nitrogen analyzer (HP 47302A, Hewlett-Packard) after mixing the expired gas. Three FRC measurements for each IAP and PEEP setting were performed and averaged.

#### Oxygenation

Arterial oxygen tension (PaO_2_) and haemoglobin concentration (Hb) were measured with a blood gas machine (ABL77, Radiometer, Copenhagen, Denmark) immediately following collection. Blood was drawn from the femoral artery and pulmonary artery in order to measure arterial, and mixed venous oxygen tensions, respectively.

#### Calculations

The following calculations were made from the measured variables: Abdominal perfusion pressure (APP) = MAP - IAP [1]. Systemic vascular resistance (SVR) = (MAP - CVP)/CO × 79.9 dyn × s/cm^5^. PaO_2 _was corrected for pH (PaO_2 _cor) = PaO_2 _x 10 (0.30 × (pH-7.4) [23]. Oxygen saturation = 100 × (0.13534 × PaO_2 _cor)^3.02 ^/((0.13534 × PaO_2 _cor)^3.02 ^+ 91.2)) [23]. Oxygen content = oxygen saturation × (%/100) × Hb (g/dl) × 1.39 (ml/g) + 0.003 (ml/dl) × PO_2 _cor) [24]. DO_2 _= CO × arterial oxygen content [24]. FRC = ((total expired gas volume × nitrogen concentration)/(100 × 0.6)) - 1.92 (measured dead space of ventilator).

#### Statistics

To detect a difference in DO_2 _of 3.0 ml/kg/min (assuming a mean (SD) DO_2 _of 18.0 (4.0) ml/kg/minute) [25] between two different PEEP values (α = 0.05, power = 80%) we calculated a sample size of 13 pigs. Data are reported as mean (SD), as the data proved to be normally distributed, when analyzed by the Kolmogorov-Smirnov test. To compare the data between the different combinations of PEEP and IAP, an ANOVA for repeated measures was performed and a *post hoc *Student-Newman-Keuls-test to adjust for multiple comparisons. A probability of < 0.05 was considered statistically significant.

## Results

**Figure 1:**
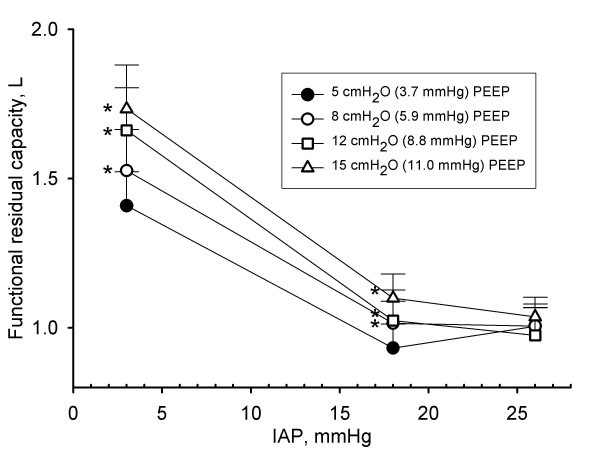
**Influence of intra-abdominal pressure and positive end-expiratory pressure on functional residual capacity**. Functional residual capacity (FRC) in litres (L) in function of different levels of intra-abdominal pressures (IAP) (3 mmHg (baseline), 18 mmHg (grade II intra-abdominal hypertension), and 26 mmHg (grade IV intra-abdominal hypertension)) at different levels of positive end-expiratory pressures (PEEP). Mean and SE are shown. ANOVA and *post hoc *Student-Newman-Keuls were used for statistical testing. *, *P *< 0.05 within an IAP setting vs. the corresponding value at 5 cmH_2_O PEEP. For clarification additional symbol is added where necessary. At each PEEP setting, all FRC values were significantly different compared to the corresponding value at baseline IAP (*P *< 0.05).

**Figure 2:**
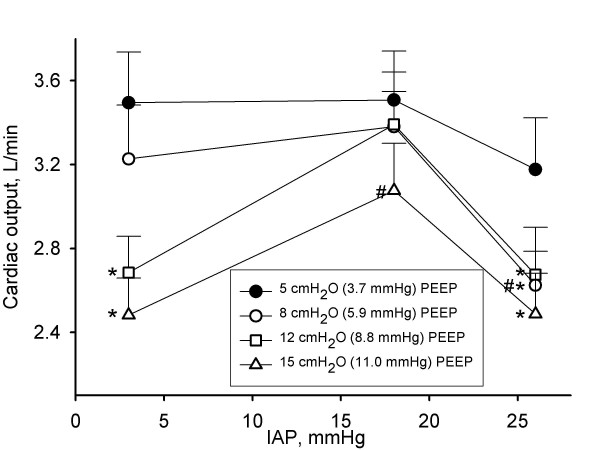
**Influence of intra-abdominal pressure and positive end-expiratory pressure on cardiac output**. Cardiac output in L/minute in function of different levels of intra-abdominal pressures (IAP) (3 mmHg (baseline), 18 mmHg (grade II intra-abdominal hypertension), and 26 mmHg (grade IV intra-abdominal hypertension)) at different levels of positive end-expiratory pressures (PEEP). Mean and SE are shown. ANOVA and *post hoc *Student-Newman-Keuls were used for statistical testing *, *P *< 0.05 within an IAP setting vs. the corresponding value at 5 cmH_2_O PEEP. #, *P *< 0.05 within a PEEP setting vs. the corresponding value at baseline IAP. For clarification additional symbol is added where necessary.

**Figure 3:**
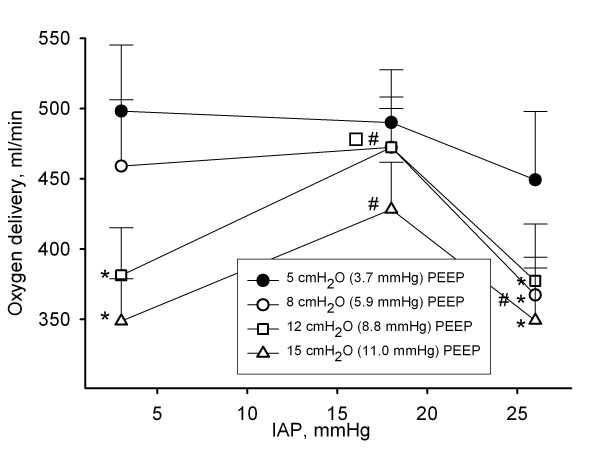
**Influence of intra-abdominal pressure and positive end-expiratory pressure on oxygen delivery**. Oxygen delivery in ml/min in function of different levels of intra-abdominal pressures (IAP) (3 mmHg (baseline), 18 mmHg (grade II intra-abdominal hypertension), and 26 mmHg (grade IV intra-abdominal hypertension)) at different positive end-expiratory pressures (PEEP). Mean and SE are shown. ANOVA and *post hoc *Student-Newman-Keuls were used for statistical testing *, *P *< 0.05 within an IAP setting vs. the corresponding value at 5 cmH_2_O PEEP. #, *P *< 0.05 within a PEEP setting vs. the corresponding value at baseline IAP. For clarification additional symbol is added where necessary.

**Figure 4:**
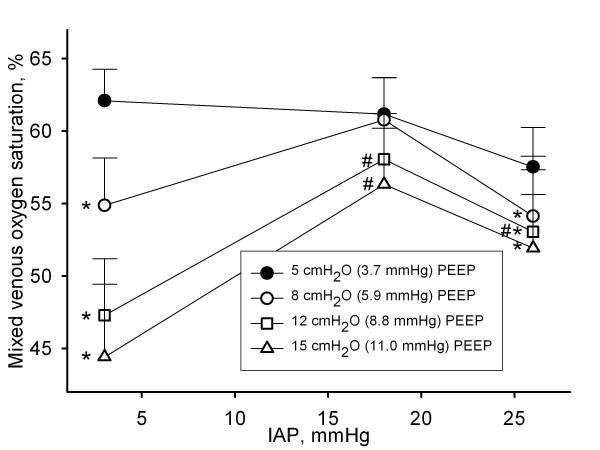
**Influence of intra-abdominal pressure and positive end-expiratory pressure on mixed venous oxygen saturation**. Mixed venous oxygen saturation in % in function of different levels of intra-abdominal pressures (IAP) (3 mmHg (baseline), 18 mmHg (grade II intra-abdominal hypertension), and 26 mmHg (grade IV intra-abdominal hypertension)) at different levels of positive end-expiratory pressures (PEEP). Mean and SE are shown. ANOVA and *post hoc *Student-Newman-Keuls were used for statistical testing *, *P *< 0.05 within an IAP setting vs. the corresponding value at 5 cmH_2_O PEEP. #, *P *< 0.05 within a PEEP setting vs. the corresponding value at baseline IAP.

Mean (SD) animal weight was 42 (8) kg. Haemoglobin concentration was 103 (8) g/L. After inflation of the intra-abdominal balloon to the target IAP, the IAP remained constant over the five-minute stabilising period. The resulting level of IAP at the time of measurement was: 3 (2), 18 (3), and 26 (4) mmHg for baseline, grade II IAH, and grade IV IAH settings, respectively. There were no differences between the measured parameters at baseline IAP and 5 cmH_2_O PEEP taken before and during the randomized protocol. An adjustment of the values according to the weight of the individual animal did not alter the findings, therefore absolute values are given. The influence of IAP and PEEP on haemodynamic and respiratory parameters is shown in Tables 1, 2 and 3, and Figures 1, 2, 3 and 4. Increasing PEEP from 5 to 15 cmH_2_O (3.7 to 11.0 mmHg) did not significantly increase IAP (+0.4 (0.8) mmHg).

**Table 1:** Influence of positive end-expiratory pressure on respiratory and haemodynamic data at baseline intra-abdominal pressure

PEEP, cmH_2_O	5	8	5 vs 8	12	5 vs 12	15	5 vs 15
FRC, L	1.4 (0.4)	1.5 (0.5)	NS	1.7 (0.5)	0.002	1.7 (0.6)	< 0.001
PaO_2_, mmHg	237 (14)	240 (19)	NS	236 (16)	NS	227 (25)	< 0.05
pPaw, cmH_2_O	21 (6)	24 (5)	< 0.001	27 (5)	< 0.001	32 (5)	< 0.001
mPaw, cmH_2_O	10 (1)	13 (2)	< 0.001	16 (2)	< 0.001	19 (1)	< 0.001
C dyn, ml/cmH_2_O	25 (8)	25 (9)	NS	24 (9)	NS	21 (6)	< 0.001
CO, L/min	3.5 (1.0)	3.2 (1.0)	NS	2.7 (0.7)	0.009	2.5 (0.7)	0.002
DO_2_, ml/min	498 (156)	459 (156)	NS	381 (112)	0.006	349 (100)	< 0.001
SvO_2_, %	62 (7)	55 (11)	< 0.05	47 (13)	< 0.05	44 (17)	< 0.05
VO_2_, ml/min	191 (47)	209 (70)	NS	205 (81)	NS	196 (53)	NS
MAP, mmHg	71 (19)	67 (15)	NS	60 (13)	0.025	56 (21)	0.004
APP, mmHg	69 (19)	64 (15)	NS	56 (13)	0.01	53 (21)	0.002
CVP, mmHg	8 (4)	8 (3)	NS	9 (2)	NS	10 (3)	NS
PAOP, mmHg	6 (2)	6 (2)	NS	8 (2)	0.004	9 (1)	< 0.001
HR, beats/min	79 (13)	81 (18)	NS	85 (20)	NS	89 (24)	0.026
SVR, dyn * s/cm^5^	1,389 (408)	1,404 (352)	NS	1,445 (373)	NS	1,337 (321)	NS
SV, ml	50 (21)	44 (13)	NS	37 (12)	0.002	33 (10)	< 0.001

APP, abdominal perfusion pressure; Cdyn, dynamic compliance; CO, cardiac output; CVP, central venous pressure; DO_2_, oxygen delivery; FRC, functional residual capacity; HR, heart rate; MAP, mean arterial pressure; mPaw, mean airway pressure; PaO_2_, arterial oxygen tension; PAOP, pulmonary artery occlusion pressure; PEEP, positive end-expiratory pressure; pPaw, peak airway pressure; SV, stroke volume; SvO_2_, mixed venous oxygen saturation; SVR, systemic vascular resistance; VO_2_, oxygen consumption. Mean (SD) are given. ANOVA and *post hoc *Student-Newman-Keuls were used for statistical testing. NS, not significant (*P *> 0.05).

**Table 2:** Influence of positive end-expiratory pressure on respiratory and haemodynamic data at 18 mmHg intra-abdominal pressure

PEEP, cmH**_2_**O	5	8	5 vs 8	12	5 vs 12	15	5 vs 15
FRC, L	0.9 (0.3) *	1.0 (0.3) *	0.034	1.0 (0.3) *	0.049	1.1 (0.3) *	< 0.001
PaO_2_, mmHg	215 (32)	218 (20) *	NS	222 (23) *	NS	216 (26)	NS
pPaw, cmH_2_O	29 (5) *	31 (5) *	< 0.001	34 (5) *	< 0.001	37 (5) *	< 0.001
mPaw, cmH_2_O	12 (3) *	15 (3) *	< 0.001	18 (3) *	< 0.001	21 (4) *	< 0.001
C dyn, ml/cmH_2_O	15 (4) *	15 (3) *	NS	16 (4) *	NS	16 (4) *	NS
CO, L/min	3.5 (0.9)	3.4 (0.7)	NS	3.4 (0.9)	NS	3.1 (0.8) *	NS
DO_2_, ml/min	490 (130)	472 (91)	NS	472 (124) *	NS	428 (116) *	NS
SvO_2_, %	61 (9)	61 (10)	NS	58 (11) *	NS	56 (14) *	NS
VO_2_, ml/min	195 (64)	192 (50)	NS	202 (55)	NS	186 (47)	NS
MAP, mmHg	83 (12)	79 (11) *	NS	81 (16) *	NS	72 (13) *	< 0.001
APP, mmHg	65 (13)	62 (12)	NS	63 (18)	NS	56 (12)	0.013
CVP, mmHg	10 (3) *	11 (2) *	NS	13 (4) *	< 0.001	15 (1) *	< 0.001
PAOP, mmHg	7 (2)	9 (1) *	< 0.001	11 (2) *	< 0.001	12 (1) *	< 0.001
HR, beats/min	73 (16)	72 (14)	NS	74 (14)	NS	76 (14)	NS
SVR, dyn * s/cm^5^	1,643 (364)	1,600 (217) *	NS	1,580 (248)	NS	1,491 (275)	NS
SV, ml	52 (23)	49 (10) *	NS	47 (14) *	NS	42 (12) *	NS

APP, abdominal perfusion pressure; Cdyn, dynamic compliance; CO, cardiac output; CVP, central venous pressure; DO_2_, oxygen delivery; FRC, functional residual capacity; HR, heart rate; MAP, mean arterial pressure; mPaw, mean airway pressure; PaO_2_, arterial oxygen tension; PAOP, pulmonary artery occlusion pressure; PEEP, positive end-expiratory pressure; pPaw, peak airway pressure; SV, stroke volume; SvO_2_, mixed venous oxygen saturation; SVR, systemic vascular resistance; VO_2_, oxygen consumption. Mean (SD) are given. ANOVA and *post hoc *Student-Newman-Keuls were used for statistical testing. *, significant (*P *< 0.05) difference compared with baseline IAP. NS, not significant.

**Table 3:** Influence of positive end-expiratory pressure on respiratory and haemodynamic data at 26 mmHg intra-abdominal pressure

PEEP, cmH**_2_**O	5	8	5 vs 8	12	5 vs 12	15	5 vs 15
FRC, L	1.0 (0.2) *	1.0 (0.3) *	NS	1.0 (0.3) *	NS	1.0 (0.2) *	NS
PaO_2_, mmHg	213 (24)	215 (21) *	NS	212 (21) *	NS	212 (23)	NS
pPaw, cmH_2_O	33 (4) *	36 (4) *	< 0.001	38 (5) *	< 0.001	42 (4) *	< 0.001
mPaw, cmH_2_O	13 (4) *	16 (4) *	< 0.001	19 (4) *	< 0.001	22 (4) *	< 0.001
C dyn, ml/cmH_2_O	13 (3) *	13 (3) *	NS	13 (4) *	NS	12 (3) *	NS
CO, L/min	3.2 (1.0)	2.6 (0.6) *	< 0.001	2.7 (0.9)	< 0.001	2.5 (0.8)	< 0.001
DO_2_, ml/min	449 (161)	367 (93) *	0.035	377 (140)	0.029	349 (124)	0.005
SvO_2_, %	58 (9)	54 (14)	0.007	53 (15) *	0.02	52 (13)	0.045
VO_2_, ml/min	188 (38)	179 (42)	NS	183 (54)	NS	165 (32)	NS
MAP, mmHg	78 (13)	74 (18)	NS	74 (15) *	NS	76 (17) *	NS
APP, mmHg	52 (14) *	48 (15) *	NS	48 (15)	NS	49 (17)	NS
CVP, mmHg	11 (3) *	12 (2) *	NS	13 (2) *	0.012	17 (3) *	< 0.001
PAOP, mmHg	9 (2)	11 (4) *	0.024	12 (2) *	0.007	14 (3) *	< 0.001
HR, beats/min	72 (10)	78 (14)	NS	76 (13)	NS	80 (16)	NS
SVR, dyn * s/cm^5^	1,771 (446)	1813 (404) *	NS	1,861 (490) *	NS	1,891 (419) *	NS
SV, ml	44 (12)	37 (14) *	0.024	39 (18)	0.037	33 (12)	0.002

APP, abdominal perfusion pressure; Cdyn, dynamic compliance; CO, cardiac output; CVP, central venous pressure; DO_2_, oxygen delivery; FRC, functional residual capacity; HR, heart rate; MAP, mean arterial pressure; mPaw, mean airway pressure; PaO_2_, arterial oxygen tension; PAOP, pulmonary artery occlusion pressure; PEEP, positive end-expiratory pressure; pPaw, peak airway pressure; SV, stroke volume; SvO_2_, mixed venous oxygen saturation; SVR, systemic vascular resistance; VO_2_, oxygen consumption. Mean (SD) are given. ANOVA and *post hoc *Student-Newman-Keuls were used for statistical testing. *, significant (*P *< 0.05) difference compared with baseline IAP. NS, not significant.

### Effect of IAP on FRC, and PaO_2 _

SaO_2 _was 99.7 (0.2)% at all levels of IAP and PEEP. IAH was associated with lower levels of PaO_2_. However, differences in PaO_2 _were only significant at 8 and 12 cmH_2_O of PEEP when comparing the differences between baseline IAP and 18 and 26 mmHg IAP (Tables 1, 2 and 3). Increasing levels of IAP were associated with a decrease in FRC by 33 (15)% and 30 (18)% for grade II and grade IV IAH, respectively (Figure 1).

### Effect of PEEP at different levels of IAP on FRC, and PaO_2 _

PEEP did not improve PaO_2 _(Tables 1, 2 and 3). The effect of PEEP on FRC varied at different levels of IAP. At baseline IAP, PEEP increased FRC (Figure 1). At grade II IAH, but not at grade IV IAH, the IAP induced FRC decline partially reversed with increasing levels of PEEP. When PEEP was increased from 5 to 15 cmH_2_O (3.7 to 11.0 mmHg), FRC increased by 0.3 (0.3) L (23 (18)%) at baseline IAP and 0.2 (0.1) L ((20 (11)%) at IAP 18 cmH_2_O.

### Effect of IAP on CO, DO_2_, SvO_2_, and SVR

IAH did not significantly change CO and DO_2 _at 5 cmH_2_O of PEEP (Figures 2 and 3, and Tables 1, 2 and 3).

### Effect of PEEP at different levels of IAP on CO, DO_2 _SvO_2_, and SVR

PEEP was associated with a dose-related decrease in CO and DO_2 _at baseline IAP and at grade IV IAH, but not at grade II IAH (Figures 2 and 3). When PEEP was increased from 5 to 15 cmH_2_O (3.7 to 11.0 mmHg), DO_2 _decreased by 151 (158) ml/minute (25 (28)%) at baseline IAP and by 100 (72) ml/minute (20 (20)%) at grade IV IAH.

The changes in SvO_2 _caused by IAH and PEEP paralleled those of CO. SVR increased significantly with rising IAP, but not with increasing PEEP.

## Discussion

There are many studies examining the influence of IAH on haemodynamic or on respiratory parameters. However, there are only a few studies investigating the effect of IAP and PEEP on cardio-respiratory parameters [26-28]. To our knowledge, this is the first study to assess the effect of different levels of PEEP in the setting of different levels of IAP on lung volumes assessed by FRC and CO parameters in a healthy pig model.

### Effect of IAP and PEEP on FRC, and PaO_2 _

We found that increasing IAP from baseline to grade II IAH decreased FRC and PaO_2 _levels by approximately 30% and 10%, respectively. There was no further decrease in FRC and PaO_2 _when IAP was increased from grade II to grade IV IAH. This suggests either a high impedance to further lengthening and cephalic motion of the diaphragm or compensatory lung expansion due to expansion of the rib cage.

Even in the absence of IAH, a healthy patient requiring mechanical ventilation will experience some degree of FRC reduction due to atelectasis [24]. Although the role of PEEP in acute lung injury and acute respiratory distress syndrome remains controversial, recruitment manoeuvres and high levels of PEEP have been shown to re-open collapsed alveoli and keep the alveoli open [17,24]. As expected in this healthy pig lung model, in the absence of IAH, PEEP increased FRC but did not increase the already high PaO_2 _levels.

In the presence of IAH, PEEP up to 15 cmH_2_O only partially reversed the IAP, induced FRC decline in grade II IAH, and did not increase FRC in grade IV IAH. PEEP did not increase PaO_2 _values in IAH.

The minimal PaO_2 _decrease as compared to the relatively larger FRC decrease in the setting of raised IAP can be explained by the FRC not dropping below the closing capacity of healthy lungs and therefore not resulting in atelectasis, shunting and consecutively impaired gas exchange [24,29]. In the setting of acute respiratory distress syndrome where the closing capacity is increased, small decreases in FRC reductions may cause marked reductions in PaO_2_. However, this would need to be confirmed in further studies.

We chose PEEP levels of 5 to 15 cmH_2_O as these represent PEEP levels frequently applied in critical ill patients. The minimal effect of PEEP on reversing the IAH induced FRC reduction can be explained by the reduced estimated trans-pulmonary end-expiratory pressures (PEEP - IAP) which would have approximated 8, -7 and -15 mmHg at PEEP of 15 cmH_2_O (11.0 mmHg) and at IAP of 3 mmHg (baseline), 18 mmHg (grade II IAH), and 26 mmHg (grade IV IAH), respectively. Therefore, with regards to improving FRC and PaO_2_, PEEP values that are equal or higher than the corresponding IAP value might be necessary to protect against IAH induced FRC and PaO_2 _decrease as has previously been suggested [13]. However, when higher PEEP levels are applied in the setting of IAH, the potential detrimental effect of high PEEP levels on CO and DO_2 _should be considered and balanced against the lowest applicable PEEP in order to avoid haemodynamic compromise in this setting [7].

### Effect of IAP and PEEP on CO, DO_2, _and SvO_2 _

In agreement with other studies [29,30], we found that PEEP caused a dose-dependent decrease in stroke volume and CO and DO_2 _(Tables 1, 2 and 3, Figures 1 and 2) which can be attributed to a reduction in venous return [29].

The effect of IAH on CO is controversial with some studies showing a decrease in CO, while other studies do not show a change or even an increase in CO in the presence of IAH [7,10-12,31]. This controversy can be explained by IAH having a biphasic and potentially opposing effect on CO which itself may be explained by the dependence of venous return on the level of IAP [7,10,31]. Low levels of IAP have been shown to increase venous return as a result of a redistribution of abdominal blood to the thoracic compartment, thus increasing stroke volume and CO [10,31]. However, further increase in IAP overcomes the compensatory effect of blood redistribution from the abdominal compartment to the thoracic compartment decreasing venous return and therefore stroke volume and CO [10,31]. In our study, IAH did not significantly reduce stroke volume, CO and DO_2 _when low levels of PEEP were applied (5 cmH_2_O, 3.7 mmHg). In agreement with other studies [7,11,12], we also found that SVR increased with rising IAP, which may be associated with a reduction in CO and DO_2_.

We found that even modest levels of PEEP depressed CO to a greater extent than IAH alone. This finding is supported by greater depression in SvO_2 _with PEEP, than with IAP (Figure 4). These findings suggest that PEEP may be detrimental by reducing DO_2 _and failing to recruit atelectatic lung. If increased levels of PEEP are indicated in the clinical setting, it might be prudent to assess CO and arterial oxygen saturation before and after increasing the level of PEEP in order to ascertain that the beneficial effect of PEEP with increasing FRC and oxygenation is not offset by a detrimental effect on CO, with a subsequent decrease in DO_2_.

However, since we used healthy lungs in our pig model, the arterial oxygen saturation was nearly 100% at all IAP and PEEP settings. Therefore, as DO_2 _is derived from arterial oxygen saturation, haemoglobin levels, and CO, the effect of PEEP and IAP on DO_2 _paralleled the effect observed on CO (Figures 2 and 3). It is important to appreciate that our findings cannot be extrapolated to patients with a failing heart, where preload and afterload are more important limitations on CO, or to patients with diseased lungs.

Grade II IAH blunted the effect of PEEP on stroke volume, CO and DO_2_. This was possibly caused by an increase in venous return associated with low levels of IAH as outlined above. Grade IV IAH did not protect against the PEEP-induced reduction in stroke volume and CO, most likely due to a reduced venous return associated with high levels of IAH [10,31]. This suggests the existence of IAP levels that are relatively resistant to PEEP induced CO reduction by counteracting the reduction in venous return caused by increasing levels of PEEP.

### Influence of PEEP on IAP

PEEP up to 15 cmH_2_O (11.0 mmHg) did not further increase IAP. Other investigators have found either absent or minimal influence of PEEP on IAP and it appears that an effect of PEEP on IAP can only be expected when PEEP approximates IAP [32-35]. Therefore our findings that PEEP did not influence IAP can be attributed to the relatively modest level of PEEP (15 cmH_2_O, 11 mmHg) in comparison to the levels of IAP (18 mmHg and 26 mmHg) used in this study (estimated trans-pulmonary PEEP of -7 and -15 mmHg, respectively).

### Limitations

We used pigs in this study because pig models have been used extensively in IAH research and the physiology of this animal is very similar to humans.

However, it is always difficult to transfer animal data into clinical practice, especially when applying higher levels of PEEP in healthy pigs with IAH. Therefore, an extrapolation of our results onto the effects of IAP and PEEP in critically ill patients remains difficult.

We used an inflatable balloon to achieve different levels of IAP as a model of acute IAH [19]. We chose not to use a pneumoperitoneum using gas inflation as used by some other investigators for two reasons. First, we wanted to eliminate the cardiovascular and respiratory response to hypercapnia when carbon dioxide or air is used when performing pneumoperitoneum [12]. Second, we wanted to measure the influence of PEEP on IAP and this is difficult to perform in the setting of a pneumoperitoneum due to possible gas leakage.

Ideally, in order to imitate the clinical setting as closely as possible, a fluid based IAH model should be used (haemorrhage, ascites, oedema). However, models using fluid instillation have their own disadvantages mainly due to uncontrollable abdominal fluid absorption with possible change in cardio-respiratory physiology [36,37].

To ensure the absence of changes in IAP caused by leakage from the balloon, we assessed the changes in IAP over time. As there were no significant changes in IAP before and after the five-minute stabilization period, we conclude that there was insignificant gas leakage from the intra-abdominal balloon or adaptive abdominal processes.

As we used healthy pigs in our experimental model it is not surprising that we obtained high PaO_2 _levels and a near 100% arterial oxygen saturation at all IAP and PEEP settings. We used a porcine mathematical model to calculate oxygen saturation that shows a good agreement with the measured oxygen saturation [23].

As we did not use an oesophageal catheter to measure pleural pressures, we are unable to give information on chest wall compliance, which is strongly influenced by IAP in the setting of IAH [33]. Trans-pulmonary pressures have been shown to be useful in titrating the level of PEEP in the setting of acute respiratory distress syndrome [38]. In the setting of IAH, trans-pulmonary pressures have been recommended not only to help titrate the level of PEEP but also to guide recruitment manoeuvres [13]. As we limited our recruitment manoeuvres to a maximum of 40 cmH_2_O airway pressure and not to maximum trans-pulmonary pressures of 25 cmH_2_O we were not able to perform sufficient recruitment in all PEEP and IAP settings, especially at 26 mmHg of IAP. This might explain the absent effect of PEEP in reversing IAP induced FRC decline in the setting of grade IV IAH, respectively. However, we think this reduced influence of PEEP in reversing IAP induced FRC decline is better explained by the relative small estimated trans-pulmonary PEEP (-7 mmHg and -15 mmHg at PEEP of 11 mmHg and IAP of 18 and 26 mmHg, respectively).

We chose four PEEP settings and three IAP settings in our experimental model, as our main focus was to study the effect of PEEP on FRC, CO and DO_2 _in the setting of increased IAP. We used PEEP values of 5, 8, 12, and 15 cmH_2_O as these PEEP values are frequently applied ventilator settings in critically ill patients. Since it remains unclear what the exact threshold value of IAP is at which a surgical abdominal decompression should be performed, we chose grade II and grade IV IAH because surgical abdominal decompression is currently not recommended for grade II whereas it is recommended for persistent grade III and IV in the presence of a new organ failure [1,2].

Another limitation is that we measured the mean IAP instead of the end-expiratory IAP as suggested by the World Society of Abdominal Compartment Syndrome [1]. As it has been shown that the difference between end-inspiratory and end-expiratory IAP increases in proportion to IAP, our measured mean IAP will underestimate end-expiratory IAP by approximately 1 mmHg at 11 mmHg end-expiratory IAP [39].

## Conclusions

The results of this experimental study show that IAH had only a minimal effect on CO and DO_2 _whereas FRC was markedly and PaO_2 _levels were minimally reduced with increasing levels of IAH. On the other hand, commonly applied PEEP levels of up to 15 cmH_2_O (11.0 mmHg) only partially restored FRC in grade II IAH and had no effect in grade IV IAH. At the same time increasing levels of PEEP may have a detrimental effect on CO and DO_2 _at high levels of IAH.

Based on these results, prophylactic PEEP levels inferior to the corresponding IAP can not be recommended in the setting of IAH as these PEEP levels are not sufficient in preventing FRC decline caused by IAH and may even be associated with a reduced DO_2 _as a consequence of a decreased CO. Further trials to assess whether higher levels of PEEP can reverse IAP induced FRC decline without impairing CO in the setting of IAH are required in the future.

## Key messages

• In this pig model, the application of commonly applied levels of PEEP (up to 15 cmH_2_O) was not able to prevent a FRC decline caused by IAH (18 mmHg and 26 mmHg).

• CO decreased with increasing levels of PEEP but not with increasing levels of IAH.

• Based on these results, prophylactic PEEP levels inferior to the corresponding IAP can not be recommended in the setting of IAH as these PEEP levels are not sufficient in preventing the FRC decline caused by IAH and may be associated with a reduced CO.

• Increasing the level of PEEP from 5 to 15 cmH_2_O did not further increase IAP in the setting of IAH.

## Abbreviations

APP: abdominal perfusion pressure; Cdyn: dynamic compliance; CO: cardiac output; DO_2_: oxygen delivery; FRC: functional residual capacity; Hb: haemoglobin concentration; IAH: intra-abdominal hypertension; IAP: intra-abdominal pressure; IV: intravenous; MAP: mean arterial blood pressure; mPaw: mean airway pressure; PaO_2_: arterial oxygen tension; PEEP: positive end-expiratory pressure; pPaw: peak airway pressure; SVR: systemic vascular resistance.

## Competing interests

The authors declare that they have no competing interests.

## Authors' contributions

AR, LH, GM, BS and PVH participated in the design of the study. AR, LH, GM, BR and BN contributed to data collection. AR performed the statistical analyses and drafted the manuscript. LH, GM, BR, BS and PVH revised the manuscript. All authors read and approved the final manuscript.

## Acknowledgements

This study was supported by the Sir Charles Gairdner Hospital Research Fund, by the Sir Charles Gairdner Hospital Intensive Care Research Fund. We thank Richard Parsons for statistical support. We thank the Department of Medical Technology and Physics as well as the team of the Large Animal Facility of the University of Western Australia for technical assistance.
